# Anxiety in the family: a genetically informed analysis of transactional associations between mother, father and child anxiety symptoms

**DOI:** 10.1111/jcpp.13068

**Published:** 2019-05-20

**Authors:** Yasmin I. Ahmadzadeh, Thalia C. Eley, Leslie D. Leve, Daniel S. Shaw, Misaki N. Natsuaki, David Reiss, Jenae M. Neiderhiser, Tom A. McAdams

**Affiliations:** ^1^ Social, Genetic and Developmental Psychiatry Centre Institute of Psychiatry, Psychology and Neuroscience King's College London London UK; ^2^ College of Education University of Oregon Eugene OR USA; ^3^ Department of Psychology University of Pittsburgh Pittsburgh PA USA; ^4^ Department of Psychology University of California Riverside, Riverside CA USA; ^5^ Child Study Center Yale University New Haven CT USA; ^6^ Department of Psychology Pennsylvania State University State College PA USA

**Keywords:** Anxiety, parent–child relationships, genetics, longitudinal, structural equation modelling

## Abstract

**Background:**

Anxiety in parents is associated with anxiety in offspring, although little is known about the mechanisms underpinning these intergenerational associations. We conducted the first genetically sensitive study to simultaneously examine the effects of mother, father and child anxiety symptoms on each other over time.

**Method:**

Adoptive parent and child symptoms were measured at child ages 6, 7 and 8 years from 305 families involved in the Early Growth and Development Study, using a prospective adoption design. Children were adopted at birth to nonrelatives, and composite data on internalising problems within birth families were used as a proxy measure of offspring inherited risk for anxiety. Structural equation models were fitted to the data to examine prospective associations between adoptive mother, father and child symptoms, whilst accounting for individuals’ symptom stability over time.

**Results:**

Child anxiety symptoms at age 7 predicted adoptive mothers’ anxiety symptoms at age 8. No mother‐to‐child or child‐to‐father effects were observed. These results were consistent in sensitivity analyses using only paternal offspring reports and using a second measure of child anxiety symptoms. Fathers’ anxiety symptoms at child age 6 prospectively predicted child symptoms, but only when paternal offspring reports were included in the model. Composite data on birth family internalising problems were not associated with child anxiety symptoms.

**Conclusions:**

Results show environmentally mediated associations between parent and child anxiety symptoms. Results support developmental theories suggesting that child anxiety symptoms can exert influence on caregivers, and mothers and fathers may play unique roles during the development of child symptoms. Further research is needed on the role of genetic transmission associated with anxiety symptoms in biologically related families. In the meantime, researchers and clinicians should strive to include fathers in assessments and consider the effects of child symptoms on caregivers.

## Introduction

It is well established that anxiety disorders run in families, with strong evidence for associations between parent and child anxiety (Lawrence, Murayama, & Creswell, 2018; Micco et al., 2009; Sydsjo, Agnafors, Bladh, & Josefsson, 2018). However, the mechanisms underlying these associations remain unclear. Children of anxious parents can inherit genes associated with the development of anxiety from their parents (a genetic mechanism); anxious parents and children can behave in ways that promote anxiety in the other (environmental mechanisms); and negative environments shared by both generations can simultaneously influence anxiety in both. These mechanisms are not mutually exclusive. To better understand intergenerational anxiety associations in families, researchers should explore multiple mechanisms concurrently, requiring the use of genetically informed, longitudinal research.

Traditional twin studies suggest that childhood anxiety symptoms are moderately heritable (Boomsma, Van Beijsterveldt, & Hudziak, 2005; Trzaskowski, Zavos, Haworth, Plomin, & Eley, 2012). This infers that around half of the individual differences in vulnerability to anxiety can be explained by environmental influences, and half by genetics. When parents and children are genetically related, it follows that any parent–child anxiety correlation might be influenced by shared genes. Researchers have used a cross‐sectional children‐of‐twins design to directly examine genetic mechanisms influencing intergenerational anxiety associations (Eley et al., 2015). Here, the correlation between parent and adolescent anxiety was not significantly confounded by genetic relatedness, indicating that the familial association could be attributable to environmental exposure to an anxious relative. However, it was not possible to identify the direction of effects between generations. Environmental parent‐to‐child anxiety transmission may occur as a result of maladaptive parenting and/or child observational and instruction‐based learning (Aktar, Nikolić, & Bögels, 2017; Ginsburg & Schlossberg, 2002). Researchers have focussed particularly on the role of parent over‐involvement (Aktar et al., 2017; Eley, Napolitano, Lau, & Gregory, 2010; Ginsburg & Schlossberg, 2002; Hudson, Comer, & Kendall, 2008; Murray, Creswell, & Cooper, 2009). Less attention has been paid to theoretical models of development which posit that intergenerational associations may be transactional (Bell, 1968). Child‐to‐parent anxiety effects have seldom been assessed. Therefore, whilst research indicates that the environment plays a significant part in the familial transmission of anxiety, our understanding of the underlying mechanisms remains limited.

To examine transactional associations between parents and children, we require longitudinal studies that enable parent‐to‐child and child‐to‐parent effects to be assessed simultaneously. The first genetically informed research on transactional effects between parent anxiety and child outcomes was conducted using the same prospective adoption sample as in the present manuscript; the Early Growth and Development Study (EGDS; Brooker et al., 2015). In the adoption design, *adoptive* parent and child associations are free from confounding by genetic relatedness. When children are adopted at birth to nonrelatives, as in the EGDS, *birth* parent and child associations act as a proxy measure of inherited genetic effects. Focussing on infant offspring in the EGDS sample, results demonstrated both parent‐to‐child *and* child‐to‐parent effects between adoptive parent anxiety symptoms and infant negative affect (Brooker et al., 2015). Like Eley et al. (2015), no evidence was found for genetic parent–child associations, using birth parent negative affect to model genetic transmission. In other samples, it has been suggested that child anxiety elicits ‘extreme control’ in mothers, with both phenotypes influenced by the *child's* genetic makeup (Eley et al., 2010); and mothers of clinically anxious children respond to child negative affect with greater intrusive involvement than mothers of nonanxious children (Hudson et al., 2008).

Given the focus on mother–child dyads in the existing literature, there are now growing efforts to investigate the role of fathers as well. A direct influence of paternal depression on offspring mental health has been reported in several (Class et al., 2012; Lewis, Neary, Polek, Flouri, & Lewis, 2017; Pemberton et al., 2010; Ramchandani et al., 2008) but not all (Tully, Iacono, & McGue, 2008) studies including fathers, which were not all genetically informed. Father–child anxiety associations have been seldom studied. Researchers suggest that mothers’ and fathers’ anxiety and behaviour may be differentially associated with offspring anxiety across development, with the father's role increasing over time (Connell & Goodman, 2002; Hudson et al., 2008; Moller, Majdandzic, & Bogels, 2015; Weijers, van Steensel, & Bögels, 2018). Evolutionary theory suggests that fathers encourage offspring to confront the external world, and children look more to fathers in threatening situations for clues on how to respond. Anxious fathers may not fulfil these roles, thereby influencing the development of child anxiety (Bögels & Perotti, 2011; Bögels & Phares, 2008; Paquette, 2004). Furthermore, as fathers typically adopt fewer care‐giving responsibilities than mothers, they may be less susceptible to the emotional impact of offspring psychopathology (Bögels & Perotti, 2011; Weijers et al., 2018). Ideally, researchers should examine the role of both parents together, considering how all three individuals influence one another's mental health (Davies & Cicchetti, 2004). Such work should assess whether previously reported dyadic parent–child anxiety associations remain significant in mother–father–child analyses, which better reflect the social and genetic nature of most families.

We used the EGDS adoption sample to conduct the first study of transactional associations between parent and child anxiety symptoms during middle childhood. We follow‐up on previous research showing that intergenerational anxiety associations between adolescent offspring and parents are under environmental influence (Eley et al., 2015). We expected to find similar results during middle childhood when anxiety disorders first begin to develop, whilst expanding on this to explore transactional intergenerational effects. We used age‐appropriate anxiety measures in each generation, with two parents reporting on child anxiety. We controlled for passive genetic effects by examining children who were adopted at birth, and we explored the role of inherited effects using a composite measure of lifetime internalising problems among birth parents and their first‐degree relatives. This composite was designed to reflect a single internalising liability factor, capturing multiple internalising symptoms and diagnoses across time, based on evidence for their strong genetic overlap and that persistent problems are under greater genetic influence (Caspi et al., 2014; Kendler et al., 2011; Krueger & Markon, 2006; Waszczuk, Zavos, Gregory, & Eley, 2014). We sought to understand whether lifetime risk reported in adulthood was associated with genetically influenced symptoms in biological offspring (Boomsma et al., 2005). We included birth parent, adoptive mother, father and child symptoms together in longitudinal models. We expected that results would differ by parent gender, but no further expectations were made as this is the first genetically informed, longitudinal study of transactional intergenerational anxiety associations.

## Method

### Participants

Participants were drawn from Cohort I of the EGDS, comprising 361 linked triads of adopted children, adoptive parents and birth parents (Leve et al., 2013). Cohort I recruitment ran from 2003 to 2006 with the help of 33 US adoption agencies across 10 states. All children were adopted domestically and placed with their adoptive families within 90 days of birth (mean age 3 days, *SD* 5 days). This study used data collected from adoptive parents when children were aged 6, 7 and 8 years, and from adult birth parents 18 and 54 months postpartum. All triads with same‐sex adoptive parents were removed, to enable comparison of mother–child and father–child dyads, leaving a final sample of 305 families (43% female offspring) who were not missing on all data.

### Ethical considerations

After receiving a complete description of their participation, all parents provided written informed consent for themselves, and adoptive parents consented for their child. Ethical approval was obtained from the three institutional review boards for the universities involved in data collection.

### Offspring anxiety symptoms

Offspring anxiety symptoms were measured via adoptive mother and father report using the Anxious/Depressed subscale of the Child Behavior Checklist (CBCL); one of the most established measures of maladaptive behaviours during childhood, demonstrating strong psychometric features among children aged 1.5–18 years (Achenbach & Rescorla, 2004). Items focus on trait‐level descriptions of anxiety, with 8 age‐appropriate items in the preschool version (used in this study at child age 6) and 13 age‐appropriate items in the school‐age version (used at child ages 7, 8). All items were assessed via a Likert scale where 1 = ‘not true’ and 3 = ‘very true’. Mean item scores were calculated for each reporter at each wave (mother α = .68–.78, father α = .65–.79), and average parent scores were used to reduce shared method variance. Parent reports correlated strongly on the preschool version and moderately on the school‐age version (age 6/7/8 *r *=* *.86/.40/.39). Where results were missing from one adoptive parent, scores from the nonmissing parent were used alone (age 6/7/8 *n *=* *40/42/65). In sensitivity analyses, offspring anxiety symptoms were assessed using adoptive mother and father reports separately, and with average mother–father scores on the Eley Anxiety Measure (described in Figure S1, Eley et al., 2003). Items in the Eley Anxiety Measure focus on clinical symptoms of anxiety disorders, in contrast to the trait‐level descriptions included in the CBCL. Average scores were moderately correlated between the two measures (age 6/7/8, *r *=* *.54/.43/.65).

### Adoptive parent anxiety symptoms

Adoptive parent anxiety symptoms were measured by self‐report when children were aged 6, 7 and 8 years using the 20‐item Trait Anxiety scale from the State‐Trait Anxiety Inventory for Adults (STAI). The STAI is among the most widely used measures of general trait‐level anxiety, demonstrating strong psychometric properties in adults (Spielberger, 1989). It is assessed against a four‐point scale where 1 = ‘almost never’ and 4 = ‘almost always’ (mother α = .91–.92, father α = .91–.92). Items corresponded with the child CBCL items assessing trait‐level anxiety, with both scales measuring self‐confidence, feelings of inadequacy, nervousness and worries.

### Offspring inherited risk

Offspring inherited risk was measured using a composite score previously designed for use in the EGDS (Marceau et al., 2015). Four indicators relating to internalising problems in the birth family were used to compute the score – derived from birth mother and father self‐reported data 18 and 54 months postpartum. For the first three indicators, the Composite International Diagnostic Instrument (Kessler & Ustun, 2004) was used to create birth parent counts for lifetime symptoms and diagnoses, and age of onset, for 11 internalising disorders (major depression, recurrent brief depression, dysthymia, separation anxiety, adult separation anxiety, social phobia, agoraphobia with and without panic, panic disorder, specific phobia and generalised anxiety). Episodes of antenatal birth mother internalising were excluded and instead used in the calculation of obstetric complications. The fourth indicator comprised the number of first‐degree relatives who had ever been diagnosed with an internalising disorder, derived from birth parent reports for each relative using the Family History‐Research Diagnostic Criteria (Endicott, Andreasen, & Spitzer, 1977). The composite score was intended to be a more robust measure of inherited anxiety risk than any single assessment, given that internalising disorders load highly onto a single liability factor and show strong genetic overlap in adulthood (Caspi et al., 2014; Kendler et al., 2011; Krueger & Markon, 2006; Waszczuk et al., 2014). Indicators were entered to a principal component analysis (PCA) separately for each disorder, and extracted factor scores were aggregated to create the total. Missing data for variables in each PCA were imputed in R (using package missMDA) for up to 22% of birth mothers and 64%–70% of birth fathers, depending on the indicator. The score explained 34% of the variance for internalising in the full EGDS sample (*N* = 551, Eigenvalue = 2.69). For the present study: *n* = 268, *M* = −0.20, *SD* = 1.39, min = −3.81, max = 5.44, skew = 0.68, kurtosis = 3.83.

### Covariates

Data on obstetric complications, adoption openness and child sex (male = 1, female = 2) were included in analyses as possible confounding variables. Birth mother reports and medical records relating to obstetric complications were collected 5 months postpartum. These data were used to calculate five indices of risk that were combined to create a weighted risk total score (Marceau et al., 2016). Adoption openness was characterised as the degree of contact, disclosure and frequency of communication between birth parents and adoptive families. Openness was measured six times from child age 5 months–6 years by birth mothers and adoptive parents using a seven‐point scale, where 1 = ‘very closed’ and 7 = ‘very open’. A mean standardised score was created at each time using data from each available parent's score (e.g. Ge et al., 2008).

### Analyses

All anxiety variables were log transformed to correct for skew. Outliers were reduced to a maximum value of three standard deviations above or below the mean. Robust maximum likelihood estimation (MLR) in Mplus 7.4 was used to fit triadic auto‐regressive cross‐lagged structural equation models to the adoptive family data, computing maximum likelihood estimates for missing data. In these models, the auto‐regressive paths account for stability over time. The cross‐lagged paths account for prospective effects from one individual to another. Birth parent data were included as a proxy measure of offspring inherited risk for anxiety. Covariates were included in all associations involving birth parent, adoptive parent and offspring data at 6 years (the earliest measurement point).

Full unconstrained models were examined and nonsignificant cross‐lag paths with the smallest standardised effect sizes were removed one‐by‐one, ensuring that model fit was not lost. Changes in model fit were assessed using chi‐square difference tests, adjusting chi‐square by the Satorra–Bentler scaling correction (SCR; as is required when using the MLR estimator in Mplus). Model fit was considered adequate if the root‐mean square error of approximation (RMSEA), comparative fit index (CFI) and Tucker–Lewis index (TLI) fell within recommended ranges (Hu & Bentler, 1999). In sensitivity analyses, models were rerun using the Eley Anxiety Measure for child symptoms, to investigate whether results were consistent across a second child construct. Next, models were rerun independently for each child informant to assess shared method variance. Results from the sensitivity analyses are included in Figures S1 and S2.

## Results

Descriptive statistics are shown in Table 1 (birth parent data described in methods). Repeated‐measures ANOVA suggested that adoptive mothers reported significantly higher levels of anxiety symptoms compared to adoptive fathers at ages 6 and 7 (6 years: *t*(556) = 2.52, *p *=* *.01; 7 years: *t*(535) = 2.37, *p *=* *.02; 8 years: *t*(398) = 1.46, *p* = .14). Fewer adoptive fathers participated compared to adoptive mothers and not all parents participated at every age, with attrition evident over time (see Table 1). By age 8, 7% of children scored within the borderline or clinical range for an anxiety disorder.

**Table 1:** Descriptive statistics for anxiety symptoms in adoptive families

Child age	*n*	Mean	*SD*	Min	Max
Adoptive Mother: (measure range 20–80)					
6 years	290	35.01	8.86	20.00	71.00
7 years	284	34.88	8.60	20.00	72.00
8 years	225	34.27	8.73	20.00	68.00
Adoptive Father: (measure range 20–80)					
6 years	268	33.14	8.63	20.00	59.00
7 years	253	33.13	8.44	20.00	57.00
8 years	175	32.95	9.34	20.00	62.00
Child: (measure range 0–16 at age 6; 0–26 at ages 7, 8)					
6 years	263	1.70	1.45	0.00	8.50
7 years	243	2.05	2.04	0.00	16.00
8 years	233	2.52	2.72	0.00	14.00

Child data represent average adoptive parent scores, using the preschool Child Behavior Checklist (CBCL) at age 6 and school‐age CBCL at 7, 8. Scores were standardised to enable longitudinal comparisons. Adoptive parent symptoms did not change significantly (mothers: *F*_2,494_ = 2.22, *p* = .11; fathers: *F*_2,411_ = 0.58, *p* = .56) but child symptoms did (*F*_2,439_ = 6.79, *p* < .001). Child anxiety differed by sex only at age 6 (*t*(261) = *−*3.04, *p *=* *.003).

Cross‐time within‐measure pairwise correlations (Table 2, white boxes) showed moderate to strong continuity over time. Child anxiety symptoms were not associated with birth parent internalising (dark grey box). Child symptoms correlated significantly with current and future adoptive mother symptoms (light grey box, left), but adoptive mother symptoms did not correlate significantly with future child symptoms. Child anxiety symptoms correlated with adoptive father symptoms on fewer instances than for adoptive mothers (light grey box, right). Adoptive father symptoms correlated significantly with future child symptoms, but child symptoms did not correlate significantly with future father symptoms.

**Table 2:** Pairwise correlations between adoptive mother, father and child anxiety symptoms and composite birth parent internalising data

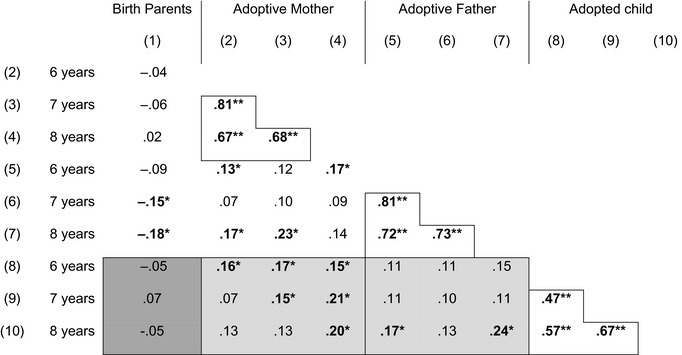

Data transformed and standardised *p < .05, ^**^ *p* < .001.

**Figure 1:**
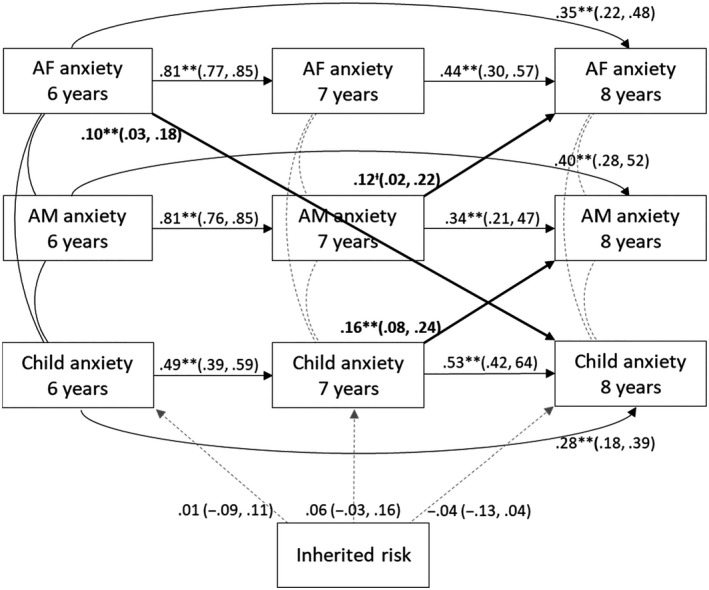
Results from the constrained structural equation model examining associations between adoptive father (AF), mother (AM) and child anxiety symptoms. Standardised parameter estimates ^Ɨ^ *p* = .05, **p < .001 (95% CI). Composite birth parent internalising data included as a proxy measure for child inherited anxiety risk. Nonsignificant cross‐lag paths are dropped, remaining nonsignificant paths shown in dashed lines. Covariates are not displayed. Model fit statistics shown in Table S2

Figure 1 shows the final model in which all nonsignificant cross‐lag paths were removed. Child anxiety symptoms at age 7 prospectively predicted adoptive mother anxiety symptoms at age 8 (*B* = .16). No significant mother‐to‐child effects were found. In contrast, adoptive fathers’ anxiety symptoms at age 6 prospectively predicted child anxiety symptoms at age 8 (*B* = .10). No significant child‐to‐father effects were found. Results suggested that anxiety symptoms in adoptive mothers at the 7‐year assessment prospectively predicted symptoms in adoptive fathers at 8 years (*B* = .12). Composite data on internalising problems among birth families were not associated with adopted child anxiety symptoms (*B* = −.07 to .06), and dropping this variable had no effect on model fit. Table S1 lists the fit indices for the constrained and unconstrained models.

Results were equivalent in bivariate models when mother–child and father–child dyads were analysed separately. Child sex was not significantly associated with adoptive parent anxiety symptoms but was significantly associated with child symptoms at age 6 (*B* = .17, 95% CI 0.08–0.27, *p* = .003). Obstetric complications and adoption openness were not significantly associated with the study variables, except for adoption openness at 5 months with birth parent composite data (*B* = .22, 95% CI 0.5–0.39, *p* = .04).

To evaluate the sensitivity of our results to alternative definitions of anxiety, we reran analyses using a different scale of mother‐ and father‐reported child anxiety (the Eley Anxiety Measure, Table S2). Child‐to‐mother, father‐to‐child and mother‐to‐father effects were found again, although the father‐to‐child effect was identified at an earlier timepoint (Figure S1). We also reran analyses using father‐only report of child anxiety, then mother‐only report. The child‐to‐mother effect remained when child symptoms were reported by fathers, but the father‐to‐child effect was not found when child symptoms were reported by mothers (Figure S2).

## Discussion

This is the first genetically informed study to assess transactional associations between parent and child anxiety symptoms during middle childhood. Using adoption data from the EGDS, we showed that child anxiety symptoms at age 7 prospectively predicted maternal, but not paternal, anxiety symptoms. This was significant in models where adoptive mother, father and child anxiety symptoms were all able to influence one another, whilst accounting for auto‐regressive effects. No mother‐to‐child effects were found. Results were consistent across two child anxiety constructs, using both maternal and paternal reports. When including paternal child reports, results suggested that paternal anxiety symptoms could prospectively predict child anxiety symptoms from age 6. We did not find an association between offspring anxiety symptoms and our composite measure for birth parents’ family history and lifetime symptoms, diagnoses and age of onset for 11 internalising disorders.

Whilst we know that intergenerational anxiety transmission is likely to be under environmental influence (Eley et al., 2015), this is the first study to show that such transmission can run from children to mothers during middle childhood. This adds to the growing body of evidence that children are active players within their environment, capable of exerting influence on their caregivers (Bell, 1968). It was reassuring to find that the child‐to‐mother effect remained consistent in sensitivity analyses: first using a separate child anxiety construct, and second when child symptoms were reported by adoptive fathers only. Previous analyses in this sample showed that infant fussing and crying predicted father anxiety (Brooker et al., 2015); however, we did not find the same for child internal symptoms during middle childhood. Our results suggest that mothers were at higher risk than fathers for being affected by offspring symptoms. Because mothers typically provide more day‐to‐day child care than fathers, they are perhaps more exposed and attuned to offspring anxiety, which can be worrying for parents (Bögels & Perotti, 2011; Hudson et al., 2008; Weijers et al., 2018). Therefore, having an anxious child may be more anxiety provoking for mothers compared to fathers. Given that mothers’ over‐involvement is associated with child anxiety (Aktar et al., 2017; Eley et al., 2010; Ginsburg & Schlossberg, 2002; Hudson et al., 2008; Murray et al., 2009), we could next explore whether these parenting behaviours develop *in response* to child anxiety and are associated with *subsequent* increases in maternal anxiety.

Like existing research on paternal depression (Class et al., 2012; Lewis et al., 2017; Pemberton et al., 2010; Ramchandani et al., 2008), we identified significant father‐to‐child anxiety effects, which were not also found for mothers. This is noteworthy given the wealth of previous research focussed on mother‐to‐child transmission, although our father‐to‐child effect was not replicated at the same ages using the Eley Anxiety Measure (Figure S1) nor when child symptoms were reported by adoptive mothers only (Figure S2). It is a strength in our research that we were able to compare perceptions of offspring anxiety across two parents, who may identify different aspects of the complex trait in their child (see Boomsma et al., 2005 for further discussion). Whilst we note that shared method variance appeared more pronounced for father compared to mother reports of self and child (Table S3), we believe that it would not provide a complete picture if fathers’ perspectives were excluded. Results derived from paternal reports of child anxiety are in line with the evolutionary theory that fathers have a potentially important role to play in guiding their child's approach to exploring the world and responding to threat (Bögels & Perotti, 2011; Bögels & Phares, 2008; Paquette, 2004). Whilst researchers have previously examined differences in the *magnitude* of mother–child and father–child psychopathology associations (Connell & Goodman, 2002; Weijers et al., 2018), we suggest that differences may lie in *directionality*. It will be of interest to explore these associations at later stages of development, considering whether results reflect diverging roles for primary and secondary caregivers (with parents able to swap between these) rather than parent gender differences. The marital relationship is also implicated in this family systems process (Bögels & Brechman‐Toussaint, 2006; Davies & Cicchetti, 2004), supported by our reported mother‐to‐father effect. We could next examine whether a transactional cycle of environmental transmission exists longitudinally, where family members repeatedly influence one another, jointly contributing to the maintenance of familial anxiety symptoms.

Nonsignificant paths with negligible effect sizes were found between child anxiety symptoms and our composite measure for internalising problems within birth families, which acted as a proxy for inherited effects. This is consistent with the two relevant studies to date, where no evidence was found for a genetic effect on intergenerational associations involving parent anxiety (Brooker et al., 2015; Eley et al., 2015). However, it is possible that these studies, along with our own, were underpowered to detect genetic effects. Equally, measures of inherited effects in adoption studies could lack validity. Although moderate heritability estimates are reported for anxiety symptoms during middle childhood (using the same measures as in this manuscript; Boomsma et al., 2005; Trzaskowski et al., 2012), it could be that the same genes influencing anxiety symptoms in children are not the same as those acting in adult parents. In support of this, longitudinal research (again using the CBCL) shows genetic innovation and attenuation on anxiety from child to adulthood (Kendler, Gardner, Annas, et al., 2008; Kendler, Gardner, & Lichtenstein, 2008; Waszczuk et al., 2014). In the present study, we aimed to maximise our chances of indexing a proxy measure of inherited risk that was related to offspring anxiety. We used a broad composite of birth parents’ family history and lifetime symptoms, diagnoses and age of onset for 11 internalising disorders, which show strong genetic overlap at both the diagnosis and symptom level with one another (Kendler et al., 2011; Waszczuk et al., 2014). When no associations were found with offspring anxiety, we explored broadening our measure with additional information on birth parent substance use and externalising, to reflect genetic risk for a single psychopathology dimension (Caspi et al., 2014). Results remained unchanged, indicating that child anxiety symptoms could not be predicted by birth parent psychopathology (results available on request). Further research experimenting with different approaches to the measurement of inherited risk will be needed, whilst also examining the possibility of moderation by familial risk (i.e. gene–environment interaction).

Strengths of this study include the longitudinal and genetically informed design, and involvement of both mothers and fathers. As in all research, there are limitations to consider. We imputed over 64% of data for birth fathers, who provide half of offspring genes but who are difficult to recruit in adoption studies. Measurement error likely reduced the association between child anxiety symptoms and our composite birth parent internalising score, especially given that this scale for inherited risk is yet to be validated. New analyses show that birth father participation in the EGDS was related to adoption openness only, not other demographic variables, and total attrition at 8 years was not related to demographic variables (Marceau et al., in press). Risk of bias due to shared method variance was increased by the attrition of more adoptive fathers than mothers, but this did not undermine the child‐to‐mother effect that remained significant when using paternal reports of child symptoms in triadic models. Since child anxiety differed by sex at age 6, the slight weighting towards male offspring in our sample (43% female) should also be considered. We do not investigate the specific environmental mechanisms that underpin the reported intergenerational associations, and we did not account for factors outside of the parent–child relationship that may also be influential. Considering the generalisability of our sample, results may be biased towards American families with high socioeconomic status (Leve et al., 2013), and adopted children who are at higher risk of experiencing prenatal adversity and inheriting genes associated with psychopathology (Cadoret, 1990). We did not find evidence for an effect of obstetric or genetic factors, although our results cannot disprove their influence. The adoption design relies on the assumption that adoption openness (controlled for in our analyses) and selective placement will have minimal effect on results. Previous EGDS research found no significant correlations for personality, cognitive and economic characteristics between birth and adoptive parents, which are unlikely to be subject to evocative gene–environment effects (Leve, Neiderhiser, Scaramella, & Reiss, 2008). Results may differ within clinical samples where parents and children are at the extreme end of the anxiety symptom distribution. Nevertheless, we provide useful information about many parents and children who experience symptoms of anxiety but who are not yet included on clinical registers. Our results correspond with a clinical study where child anxiety influenced mothers but not fathers (Hudson et al., 2008).

To our knowledge, this is the first genetically informed study to test the theoretical hypothesis that both parents and children can be implicated in perpetuating intergenerational anxiety associations. We present novel evidence for an environmentally transmitted, prospective effect of child anxiety symptoms on mothers’ symptoms during middle childhood. We suggest that parents and children may influence each other in different ways, with preliminary evidence that children are more susceptible to the effects of fathers’ than mothers’ symptoms. Further research aiming to replicate our findings across methodologies and populations will help to validate these results, whilst further investigating the role of genetic transmission between biologically related parents and children. In the meantime, researchers and clinicians should strive to include fathers in assessments and consider the effects of child symptoms on caregivers, to better understand the intergenerational transmission of anxiety symptoms.

**Key points**

- Anxiety disorders are known to run in families; however, little is known about how this happens.
- We compared children who were adopted at birth to both their biological and adoptive parents, to explore genetic and environmental anxiety transmission in families during middle childhood.
- Child anxiety symptoms at age 7 predicted adoptive mothers’ symptoms at age 8 in all analyses. Adoptive fathers’ symptoms predicted child symptoms if paternal offspring reports were included in the model.
- Child anxiety symptoms were not associated with the proxy measure for inherited risk – which indexed birth parents’ family history and lifetime symptoms, diagnoses and age of onset for 11 internalising disorders.
- Clinicians should include fathers in assessments and consider the effects of child symptoms on caregivers, to better understand the environmental transmission of familial anxiety.

## Supporting information


**Figure S1.** Results from the first sensitivity analysis, with child anxiety measured using the Eley Anxiety Measure.
**Figure S2.** Results from the second sensitivity analysis, with child anxiety measured using (a) father and (b) mother reports separately on the CBCL anxious/depressed subscale.
**Table S1.** Model fit indices for the unconstrained and constrained structural equation models.
**Table S2.** Descriptive statistics for the Eley Anxiety Measure.
**Table S3.** Pairwise correlations between adoptive parent and adopted child anxiety symptoms, across four indices of child anxiety symptoms (by measure and parent reporter).
