# Supplementary material for: Anxiety in the family: a genetically informed analysis of transactional associations between mother, father and child anxiety symptoms
Source: J Child Psychol Psychiatry. 2019 May 20;60(12):1269–77. doi: 10.1111/jcpp.13068 (PMC6856374; doi:10.1111/jcpp.13068)
Supplement: Supplementary file 1 — Figure S1. Results from the first sensitivity analysis, with child anxiety measured using the Eley Anxiety Measure. Figure S2. Results from the second sensitivity analysis, with child anxiety measured using (a) father and (b) mother reports separately on the CBCL anxious/depressed subscale. Table S1. Model fit indices for the unconstrained and constrained structural equation models. Table S2. Descriptive statistics for the Eley Anxiety Measure. Table S3. Pairwise correlations between adoptive parent and adopted child anxiety symptoms, across four indices of child anxiety symptoms (by measure and parent reporter). [file JCPP-60-1269-s001.docx]

**Supporting Information – Anxiety in the family: A genetically informed analysis of associations between mother, father and child anxiety symptoms – by Ahmadzadeh et al.**

**Figure S1.** Results from the first sensitivity analysis, with child anxiety measured using the Eley Anxiety Measure^a^.

Figure shows the constrained structural equation model, examining associations between adoptive-father (AF), -mother (AM) and child anxiety symptoms. Standardised parameter estimates *p<.05, **p<.001 (95% CI). Composite birth parent internalising data included as a proxy measure for child inherited anxiety risk. Non-significant cross-lagged paths are dropped, remaining non-significant paths shown in dashed lines. Covariates are not displayed. Model fit statistics shown in Table S1. Mother-child results remained consistent. A father-to-child path was found, although this did not replicate the primary findings in the paper.


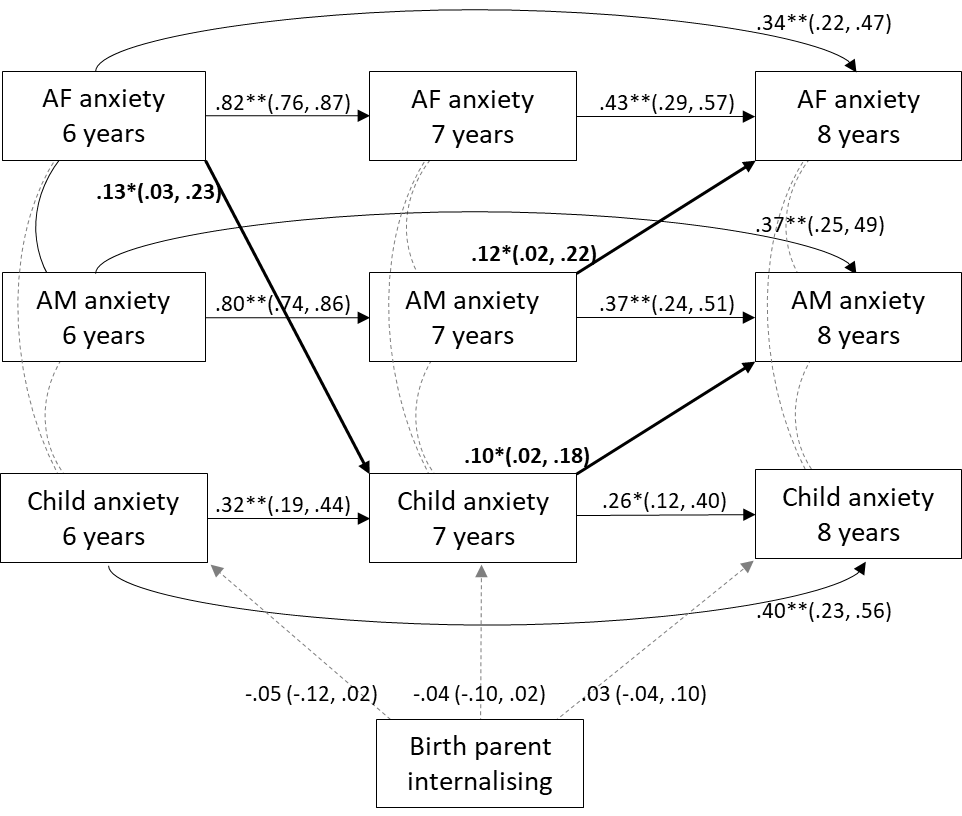


^a^ The Eley Anxiety Measure ([Eley et al., 2003](#_ENREF_15)) comprises 25 items in 6 subscales that reflect the major anxiety diagnoses and temperamental contribution to anxiety-related behaviours (general distress; separation anxiety; fear; obsessive compulsive behaviours; shy and inhibited behaviors; and poor self-esteem). Previously researchers have shown that items on this scale can distinguish between differing aspects of anxiety-related behaviours in children as young as 4 years ([Eley et al., 2003](#_ENREF_15)). As with the CBCL, items were assessed via a three-point Likert scale and average mean scores from adoptive mothers and fathers were calculated (mother α=.93-.95, father α=.95-.96; parent report correlations as age 6/7/8 *r*=.26/.17/.29). Descriptive statistics are listed in Table S2.

**Figure S2.** Results from the second sensitivity analysis, with child anxiety measured using **(a)** father and **(b)** mother reports separately on the CBCL Anxious/Depressed subscale.

Figures show the constrained structural equation models, examining associations between adoptive-father (AF), -mother (AM) and child anxiety symptoms. Standardised parameter estimates *p<.05, **p<.001 (95% CI). Composite birth parent internalising data included as a proxy measure for child inherited anxiety risk. Non-significant cross-lag paths are dropped, remaining non-significant paths shown in dashed lines. Covariates are not displayed. Model fit statistics shown in Table S1. Mother-child results remained consistent, but a significant father-to-child effect was only found when using paternal reports of child anxiety symptoms.

**(a)**

**
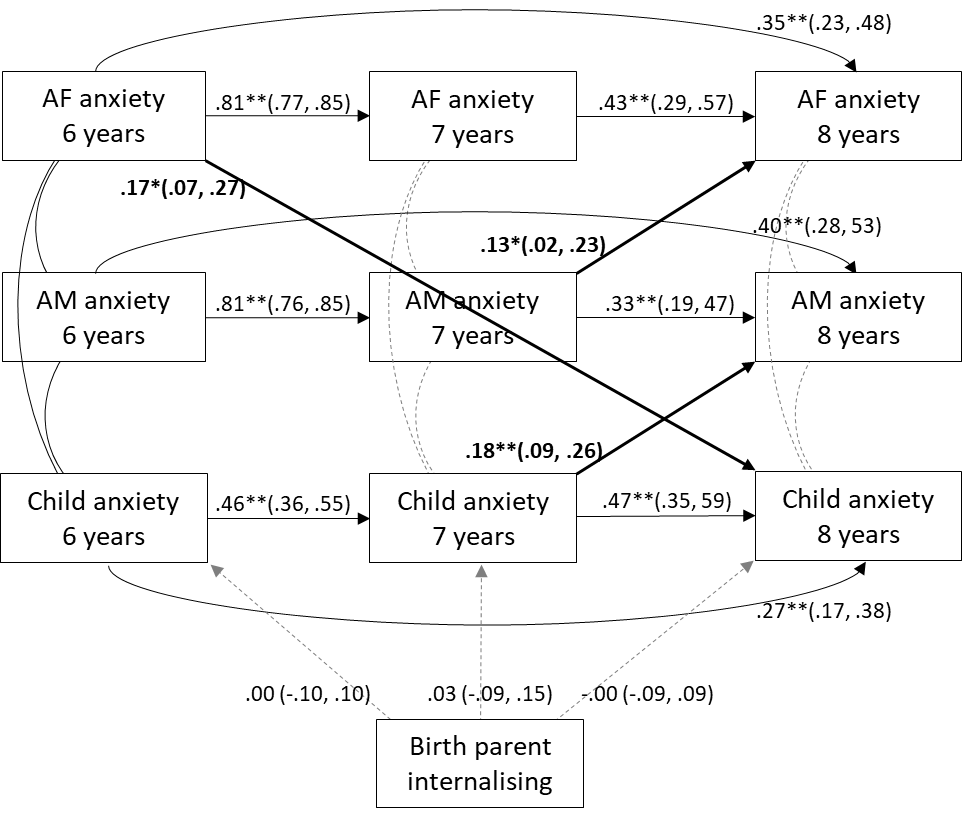
**

**(b)**

**
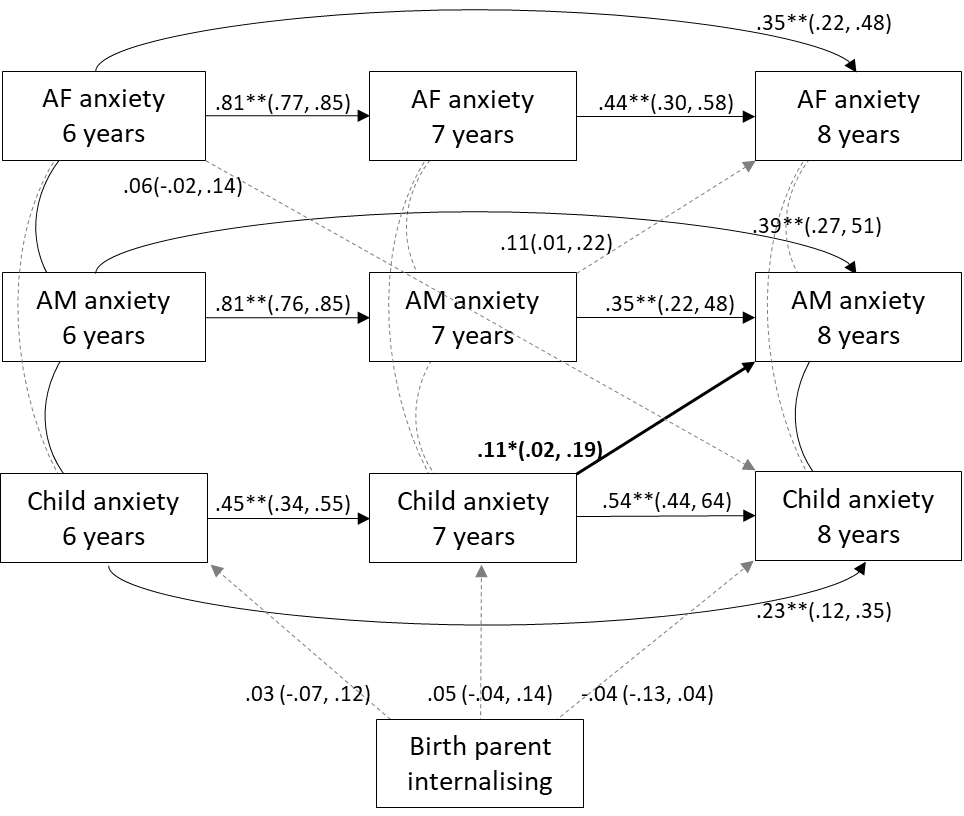
**

**Table S1.** Model fit indices for the unconstrained and constrained structural equation models.

All paths were freely estimated in the unconstrained models. All non-significant cross-lag paths were constrained in the final models.

| **Model** | ***df*** | **χ ^2^** | **p** | **RMSEA (90% CI)** | **CFI** | **TLI** | **SCR** |  |
| --- | --- | --- | --- | --- | --- | --- | --- | --- |
| ***Primary analysis*** |  |  |  |  |  |  |  |  |
| Child symptoms measured by CBCL Anxious/Depressed subscale | | | | | | | | |
| Unconstrained | 73 | 52.74 | 0.96 | <.001 (.000-.000) | 1.00 | 1.03 | 1.01 |  |
| Constrained | 88 | 59.40 | 0.99 | <.001 (.000-.000) | 1.00 | 1.04 | 1.02 |  |
| ***Sensitivity analysis 1*** | | | | | | | |  |
| Child symptoms measured by Eley Anxiety Measure | | | | | | | | |
| Unconstrained | 73 | 51.00 | 0.98 | <.001 (.000-.000) | 1.00 | 1.04 | 1.00 |  |
| Constrained | 88 | 57.13 | 0.10 | <.001 (.000-.000) | 1.00 | 1.05 | 1.01 |  |
| ***Sensitivity analysis 2*** | | | | | | | |  |
| Child symptoms measured by CBCL Anxious/Depressed subscale, adoptive father report | | | | | | | |  |
| Unconstrained | 73 | 53.81 | 0.96 | <.001 (.000-.000) | 1.00 | 1.03 | 1.02 |  |
| Constrained | 88 | 69.30 | 0.93 | <.001 (.000-.010) | 1.00 | 1.03 | 1.01 |  |
| Child symptoms measured by CBCL Anxious/Depressed subscale, adoptive mother report | | | | | | | |  |
| Unconstrained | 73 | 56.48 | 0.92 | <.001 (.000-.011) | 1.00 | 1.03 | 1.02 |  |
| Constrained | 88 | 62.98 | 0.98 | <.001 (.000-.000) | 1.00 | 1.04 | 1.02 |  |
| RMSEA (90% CI)= Root-Mean Square Error of Approximation (90% confidence intervals) | | | | | | | | |
| CFI = Comparative Fit Index | | | | | | | | |
| TLI = Tucker Lewis Index | | | | | | | | |
| SCR = Satorra-Bentler Scaling Correction | | | | | | | | |

**Table S2.** Descriptive statistics for the Eley Anxiety Measure.

Child anxiety did not change significantly over time (F_2,451_=1.56, p=.21) and reports did not differ significantly by sex at any age (t(261)=-.67, p=.51; t(253)=-.59, p=.56; t(231)=-.65, p=.52).

|  | *n* | Mean | SD | Min | Max |
| --- | --- | --- | --- | --- | --- |
| Child: (measure range 0-8) | | | | | |
| 6 years | 263 | 0.92 | 1.00 | 0.00 | 7.83 |
| 7 years | 255 | 0.96 | 1.10 | 0.00 | 7.67 |
| 8 years | 233 | 1.11 | 1.28 | 0.00 | 8.00 |

**Table S3.** Pairwise correlations between adoptive parent and adopted child anxiety symptoms, across four indices of child anxiety symptoms (by measure and parent reporter).

Data transformed and standardised *p<.05, **p<.001.

| Adopted Child | | Adoptive Mother | | | Adoptive Father | | |
| --- | --- | --- | --- | --- | --- | --- | --- |
|  |  | 6 years | 7 years | 8 years | 6 years | 7 years | 8 years |
| CBCL, Anxious Depressed: Mother report | 6 years | **0.19**** | **0.17**** | 0.13 | 0.00 | 0.00 | 0.03 |
|  | 7 years | 0.09 | **0.13*** | **0.16*** | -0.01 | -0.02 | 0.00 |
|  | 8 years | 0.13 | **0.17**** | **0.26**** | 0.04 | 0.02 | 0.12 |
| CBCL, Anxious Depressed: Father report | 6 years | **0.16**** | **0.17**** | **0.15*** | 0.11 | 0.11 | 0.15 |
|  | 7 years | 0.06 | **0.16*** | **0.23**** | **0.21**** | **0.23**** | **0.21**** |
|  | 8 years | 0.08 | 0.05 | 0.06 | **0.27**** | **0.21**** | **0.29**** |
| Eley Anxiety Measure: Mother report | 6 years | **0.15*** | 0.13 | 0.11 | -0.05 | 0.00 | -0.06 |
|  | 7 years | 0.12 | **0.17**** | **0.16*** | 0.03 | 0.02 | 0.10 |
|  | 8 years | 0.08 | 0.11 | 0.13 | -0.03 | -0.01 | 0.01 |
| Eley Anxiety Measure: Father report | 6 years | 0.10 | 0.08 | **0.16*** | **0.23**** | **0.20**** | **0.28**** |
|  | 7 years | 0.05 | 0.06 | 0.13 | **0.27**** | **0.21**** | 0.16 |
|  | 8 years | 0.03 | 0.01 | -0.02 | **0.17*** | 0.10 | **0.24**** |
